# Adaptive Ridge Regression for Rare Variant Detection

**DOI:** 10.1371/journal.pone.0044173

**Published:** 2012-08-28

**Authors:** Haimao Zhan, Shizhong Xu

**Affiliations:** Department of Botany and Plant Sciences, University of California Riverside, Riverside, California, United States of America; University of Texas School of Public Health, United States of America

## Abstract

It is widely believed that both common and rare variants contribute to the risks of common diseases or complex traits and the cumulative effects of multiple rare variants can explain a significant proportion of trait variances. Advances in high-throughput DNA sequencing technologies allow us to genotype rare causal variants and investigate the effects of such rare variants on complex traits. We developed an adaptive ridge regression method to analyze the collective effects of multiple variants in the same gene or the same functional unit. Our model focuses on continuous trait and incorporates covariate factors to remove potential confounding effects. The proposed method estimates and tests multiple rare variants collectively but does not depend on the assumption of same direction of each rare variant effect. Compared with the Bayesian hierarchical generalized linear model approach, the state-of-the-art method of rare variant detection, the proposed new method is easy to implement, yet it has higher statistical power. Application of the new method is demonstrated using the well-known data from the Dallas Heart Study.

## Introduction

Over the past decade, many causal polymorphic variants for common diseases have been successfully identified by genome-wide association studies (GWAS) [1]–[3] which are based on the common-disease-common-variant (CDCV) assumption. The associated variants greatly facilitate understanding of the genetic basis underlying common diseases. However, most association studies are used to identify common variants which have minor allele frequency (MAF) greater than 1%. This is mainly because traditional SNP genotyping arrays only capture variants with relatively high MAF. Although many genetic variants have been identified for common diseases, large proportion of the heritability of a trait cannot be explained by the detected variants. Many factors can lead to the missing heritability phenomenon: (1) underestimation of the effects of common variants, (2) undetectable common variants with small effects, and (3) rare variants [4], [5]. The advance in sequencing technologies makes it possible to sequence some important candidate genes [6], [7] and even the whole genome [8]. The available sequence information allows us to find out most variants across the genome and identify associated variants with different allele frequencies.

Meanwhile, it is found that genetic variants with low MAF, often called rare variants, may substantially contribute to phenotypic expression [9]–[14]. The missing heritability phenomenon in GWAS may be due to rare variants which are not captured by the traditional SNP genotyping arrays [5]. Therefore, identifying rare variants would help understand the genetic basis and disease etiology. Rare variants, which have lower minor allele frequencies compared to common variants, tend to have larger effects than common variants [9]. Many GWAS studies indicate that most identified common variants have odds ratio ranging from 1.1 to 1.3 with a mean odds ratio of 1.36. However, the mean odds ratio of rare variants is 3.74 and most rare variants have much greater odds ratio than common variants [9]. In addition, many non-synonymous rare mutations from exon sequencing are functional variants for some common diseases [9]. Many studies have been carried out to investigate the effects of rare variants by sequencing exons of candidate genes [6], [7], [11], [12], [15] and several rare variants have been found to be associated with common diseases. For example, rare variants in the IFIH1 gene were found to be strongly associated with Type I diabetes [15]. Some rare variants in ANGPTL3 and ANGPTL5 are much more common in the lowest quartile of plasma triglyceride level [7].

Statistical power of genetic variant identification depends on the sample size, the effect of the variant and the minor allele frequency [13], [16]. Since the minor allele frequencies (MAF) of rare variants are extremely low (less than 1%∼5%), it is extremely challenging to identify the causal rare variants by using methods of traditional association studies [16]–[20]. The univariate tests (e.g. Chi-Squared test, Fisher's exact test, linear regression) have to take into account multiple test corrections to control family-wise error rate (FWER) and false discovery rate (FDR). Multiple-marker tests (e.g. multiple regression, Hotelling's 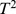 test) increase the degree of freedom in hypothesis testing. Univariate test and multivariate test both loose power when the allele frequencies are very low [18].

Until recently, much effort has been placed on the development of new statistical methods for detecting rare variants. Most of the existing methods pool variants in the same group into one variant, which collectively combines the information from multiple variants and tries to increase the power of rare variant identification. For example, cohort allelic sum test (CAST) [21] combines the rare variants in the same region into a single “variant”. The frequency of the pooled single variant can be compared between the case and control populations. The combined multivariate and collapsing (CMC) method [18] collapses rare variants in the same group into one single “variant”, and then counts the number of individuals carrying this marker in the case and control populations. The Hotelling's 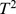 test is used to analyze the collapsed genotype data. The authors proved that the CMC method is much more powerful than the traditional single marker and multiple marker tests. It is also robust to the misclassification of rare variants. Morris and Zeggini [22] proposed two likelihood ratio tests (RVT1 and RVT2) based on different linear regression models to analyze rare variants. The first model treats the proportion of rare variants carrying at least one minor allele as predictor variable. In the second model, the indicator of presence or absence of the minor allele at any rare variant for one individual is used as the predictor in the linear model, which is similar to the collapsing method proposed by Li and Leal [18]. Madsen and Browning [19] weighed each rare variant by the minor allele frequency in unaffected individuals and found that this weighted approach can magnify the signal of rare variants. In that analysis, each individual was assigned a genetic score and the scores were ranked to test the significance of the association signal. Price et al. [23] adopted a variable-threshold approach to analyzing rare variants. They calculated a z-score for each reasonable threshold and find the maximum z-score. The significance of the z value was then tested using a permutation analysis.

All these so called burden test methods assume that the effects of rare variants are in the same direction. They collapse rare variants into a single variant and then compare the frequencies in the case and control populations. However, it is well known that genetic variants may not have effects on the phenotype of interests, and some of the variants may have beneficial effects and others have deleterious effects. Therefore, such assumptions seem to be inappropriate and collapsing rare variants in this way will introduce noise and decrease the power. Taking into account different directions of the variants will increase the power of rare variant detection [24], [25].

More recently, Yi and Zhi [20] proposed a Bayesian hierarchical generalized linear model (BhGLM) to analyze rare variants without assuming known directions of the variant effects. They introduced two types of parameters to the regression model, a weight parameter for each variant and an overall effect for all variants in the same functional unit. The weights and the overall effect are estimated using the weighted least squares method that incorporates hierarchical prior information. As a result, the weights are estimated based on the contribution of the variants to the phenotype of interest. The association between rare variants and the phenotype of a target trait can be found by testing the significance of a single parameter, the overall effect (or score). Yi et al. [26] eventually proposed a similar method based on the BhGLM. The new method incorporates covariates and divides rare variants into several groups according to the minor allele frequencies and the functions of the variants. They also assigned hierarchical prior distributions to the weights of the variants and the groups. The association between the phenotype and the variants in the same group can be found by testing the group effects. These two methods do not assume known directions of the effects of the rare variants and can identify the collective effects of rare variants in the same group as well as individual variant effects. The BhGLM has a higher power than all the burden test methods. This new method is considered the state-of-the-art method for rare variant detection.

We believe that the high power of the BhGLM is due to (1) appropriate combination of the individual rare variants (the new score) and (2) assignment of the hierarchical priors. After a thorough evaluation of these new methods, we found that there is still some room for improvement. The new score of the BhGLM method is a first moment parameter (shared effect). An alternative score may be a second moment parameter (shared variance). The Yi and Zhi's method requires prior distributions, and thus different priors may produce different results. The hyper-parameters involved in the prior distributions may also affect the results. In this study, we proposed to use a shared variance among rare variants as the new score. The method is originally called ridge regression [27]. It is further modified to discriminate against rare variants with small effects. This modified ridge regression is called the adaptive ridge regression [28]. The adaptive ridge regression (ARR) is performed under the maximum likelihood framework, and thus prior distributions of parameters are not required, equivalent to independent uniform priors for all parameters.

## Methods

The key issue in rare variant detection is to combine all rare variants into a single score (shared feature) and perform a single test for the collective effect of all rare variants. Our new method will be developed based on this notion. For the paper to be self-contained, we will briefly introduce ridge regression [27], based on which a new method called adaptive ridge regression will be developed.

### Ridge regression

Let 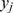 be the phenotypic value of a quantitative trait measured from individual *j* for 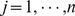, where 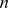 is the sample size. The following linear model is used to describe the relationship between 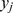 and the rare variants,
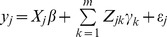
(1)where 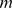 is the number of rare variants, 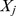 is a row vector representing the incidences of some covariates (fixed effects), 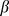 is a column vector for the fixed effects, 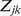 is a genotype indicator variable for marker *k*, 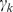 is the effect of the *k*th rare variant, and 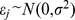 is the residual error with an assumed normal distribution. The genotype indicator variable is coded as
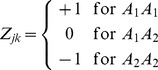
(2)where 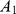 is the rare allele and 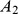 is the common allele of locus *k.* This coding system is adopted from that of QTL mapping [29]. An alternative coding system is
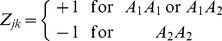
(3)where a genotype carrying one or two rare alleles is coded as 1 and the common allele homozygote is coded as −1. Because of the rare frequency of allele 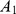, the probability of 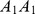 is negligible and thus the population virtually consists of only the heterozygote and the homozygote of the common allele. The second coding system has an advantage of saving computer storage. In this study, we took the first coding system, i.e., equation (2).

We assume that the effect of each rare variant is sampled from a normal distribution with mean zero and a common variance, i.e., 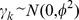 for 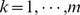. By doing this, we can evaluate the shared nature of the rare variants, i.e., they all come from the same distribution with the same mean and the same variance, so that a test statistic can be derived to test this single variance. If 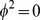, none of the rare variants are associated with the trait of interest. This particular formulation treats the cofactors as fixed effects and the rare variants as random effects. If 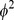 is a predetermined constant, the method is called ridge regression analysis with a ridge factor [27] of 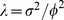. However, we can estimate 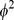 from the data under the mixed model framework [30], in which the expectation is 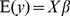 and the variance covariance matrix of the phenotypic values is
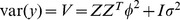
(4)where 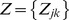 is an 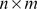 matrix for the genotype indicators of all subjects for all rare variants. The maximum likelihood or restricted maximum likelihood methods can be used to estimate the parameters 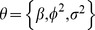 and test 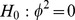. Rejection of 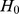 leads to a conclusion that these rare variants are collectively associated with the trait. The likelihood ratio test statistic may be used to test 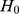,
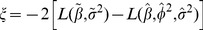
(5)where 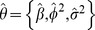 represents the ML estimate of 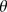 under the full model and 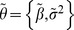 represents the ML estimate of 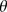 under the null model. When the sample size is sufficiently large, 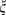 follows approximately a chi-square distribution with one degree of freedom (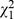). Crainceanu and Ruppert [31] stated that 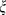 follows asymptotically a mixture of two chi-square distributions, denoted by 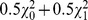. We will use both distributions to draw the critical values for the test statistic. In addition, we will also use permutation tests [32] to find the empirical distribution of the likelihood ratio test statistic and thus the empirical threshold for the controlled Type I error rate. Therefore, the asymptotic chi-square distributions may not be required in real data analysis.

The whole purpose of rare variant analysis is to test all the rare variants collectively using the likelihood ratio test statistic for 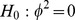. However, each individual rare variant can also be estimated and tested using the mixed model equation [30] with 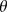 substituted by 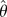. The mixed model equation is
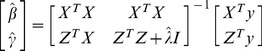
(6)where 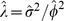. Let us define a *C* matrix by



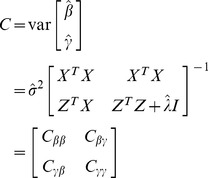
(7)The sub matrix 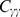 is the variance matrix for all the rare variants. Given the estimated 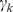 and its estimation error 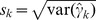, a test statistic can be drawn,
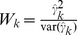
(8)from which a *p*-value can be found, assuming that (under the null model) 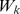 is approximately distributed as a chi-square variable with one degree of freedom. Again, in rare variant detection, our main purpose is to test 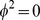, and thus testing an individual rare variant is only a by-product of the analysis.

Under the ridge regression method, each rare variant is treated as a random variable. The shared feature is the common variance denoted by 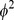, This particular treatment has eliminated the assumption of same direction of rare variant effects in all the burden test methods described in the introduction.

### Adaptive ridge regression

If only a few variants are associated with the trait, then 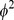 will be “diluted” by those non-associated variants. This means that the assumption of a common variance is violated. We now propose an adaptive ridge regression to selectively weigh each rare variant. The modified model is formulated as
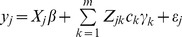
(9)where 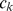 is another effect for variant *k*. It seems redundant to define two effects for each rare variant. However, the two effects, 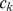 and 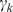, have different meanings. The first effect 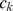 may be defined as a random effect with a marker specific variance, i.e., 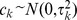 for 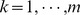. The second effect 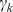 is defined as a random effect with a common (shared) variance, i.e., 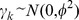 for 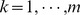. Because 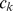 and 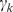 have different variances, they can be estimated separately. This partitioning of the rare variant effect appears to be similar to the model proposed by Yi and Zhi [20] but they differ in a fundamental way. Using our notation, their model can be expressed as
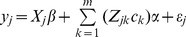
(10)in which the authors proposed a common effect 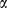 (a single first moment parameter) for all variants. We proposed marker specific effect 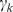 but with a common variance 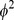. Our model is more like the polygenic model for quantitative traits, where each 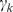 is a polygenic effect with a common genetic variance 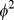.

We now discuss parameter estimation for the new adaptive ridge regression method. The method is based on the classical mixed model methodology. Given the value of each 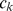, we can rewrite the adaptive ridge regression model as
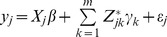
(11)where 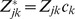 is a weighted independent variable. We can estimate 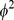 and predict 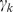 under the original ridge regression procedure (mixed model methodology) described in the previous section with 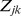 substituted by 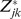. Hypothesis test for 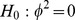 can also be performed under the mixed model framework. Each individual marker effect is actually redefined as 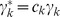 and the Wald test statistic remains the same as described before,



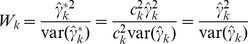
(12)The question left is how to find 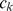 so that the adaptive ridge regression can selectively adjust 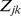 to prevent 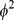 from being diluted by the non-associated rare variants. There are many different ways to estimate 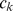. For example, we may use an iterative approach to estimating 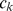 given 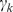 by reformulating the model as
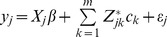
(13)where 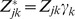 is a newly weighted independent variable. Given 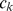 to estimate 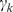 and given 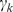 to estimate 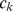 require iterations. The iteration process continues until the sequence converges. Estimating 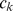 given 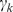 using this approach may be complicated because each 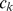 has its own variance. For *m* markers, we need to estimate *m* marker specific effects 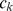 and *m* marker-specific variances 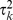 for 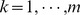. We will leave this approach as a next project and pursue a simple method to estimate 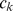.

In this study, we adopted a different method for estimating 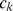. This approach leads to the Lasso [33] estimate of 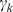 if 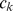 is restricted in a special way. Grandvalet [28] demonstrated that if we let 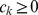 and enforce the following constraint
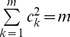
(14)the solution for 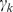 is the Lasso (least absolute shrinkage and selection operator) estimate of 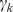, assuming that 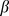 is removed from the model by centralization of the data and 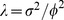 is a constant provided by the investigator prior to the analysis. In our problem, we also estimate 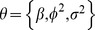 using the mixed model methodology. The Lasso parameter is estimated as a by-product because 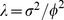 is estimated from the data, not predetermined by the investigator. Whether such a solution of 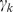 is still Lasso or not is unknown. Any way, we adopted the approach of Grandvalet [28] to find 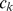 given 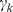, which is



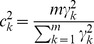
(15)During the iteration process, if any 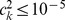 happens, 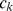 is set to zero and the corresponding 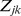 will be deleted from the model permanently so that the dimension of the model will be quickly reduced to the number of non-zero elements of vector 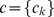. The method is not sensitive to the threshold. We tested several other thresholds, e.g., 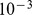 and 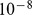. The results are much the same (data not shown). In fact, we do not need to set this threshold. The purpose of using this threshold is to improve the computational speed, because once an effect is set to zero, this effect will no longer be evaluated in the subsequent iterations.

### Adaptive ridge for multiple groups of rare variants

The model can be extended to handle multiple groups of rare variants. The notation become complicated so we have to use a compact matrix notation for the model. For a single group, we may use
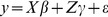
(16)where 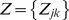 is an 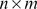 matrix and 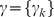 is an 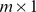 vector. Assume that we now have *g* groups and the number of markers in the *l*th group is 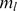, where 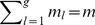 is the total number of markers. We now label the appropriate matrices by a subscript *l* to indicate the group, the extended model becomes
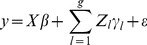
(17)where 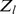 is an 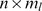 matrix and 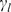 is an 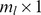 vector. The group specific 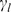 is assumed to be 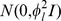 distributed. The variance matrix is



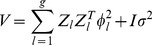
(18)The parameter vector is 
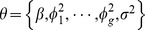
, which can be estimated using the maximum likelihood method [34], [35]. Once 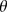 is estimated, the effects of rare variants are estimated using the mixed model equations. When 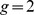, for example, the mixed model equations are
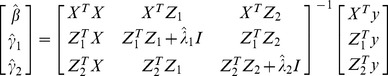
(19)where 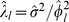 for 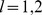. We now have 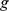 different weight systems, one for each group. Let 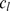 be an 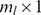 vector of weights for group 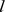. It is defined by
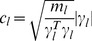
(20)where 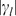 is an 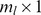 vector of the absolute values of 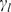 because of the constraint 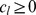. The weighted Z matrix is defined as 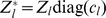, where 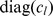 is a diagonal matrix with the diagonal elements filled by the values of vector 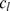. The adaptive ridge regression for multiple group rare variants is performed simply with 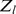 substituted by 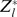.

Many different hypotheses can be tested for the multiple group variant analysis. An overall hypothesis is 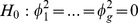, which can be tested using the likelihood ratio test statistics. Under the null hypothesis, the test statistic follows asymptotically a chi-square distribution with *g* degrees of freedom. Each individual group can also be tested. For example, to test the *l*th group, one needs to evaluate a reduced model by excluding 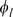 from the model and defining a likelihood ratio test statistic. Under the null hypothesis 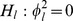, the test statistic follows asymptotically a chi-square distribution with one degree of freedom. In this study, we used the permutation test to draw the empirical threshold values of the likelihood ratio test statistics for a given Type I error rate. As a result, the asymptotic chi-squares critical values are not used in real data analysis.

## Results

### Application to the Dallas Heart Study Data

Angiopoietin-like proteins (*ANGPTLs*) [36]–[40] can regulate triglyceride metabolism by inhibiting the activity of lipoprotein lipase. Romeo et al. [6], [7] resequenced the exons and some intronic regions of *ANGPTL3* (MIM 604774), *ANGPTL4* (MIM 605910) and *ANGPTL5* (MIM 607666) genes in 3,551 individuals from a multiethnic population (601 Hispanic, 1,830 African American, 1,045 European American and 75 others). They wanted to find the sequence variants which have effects on the regulation of plasma triglyceride level. For the three genes, *ANGPTL3*, *ANGPTL4* and *ANGPTL5*, a total of 282 sequence variants (SNP) were genotyped (88 variants in *ANGPTL3*, 94 variants in *ANGPTL4* and 100 variants in *ANGPTL5*). In addition to triglyceride level and race, gender and age were also recorded for each individual. To test the effects of sequence variants, age, gender and race were treated as covariates in the adaptive ridge regression analysis. Since there are some missing data in age, all missing values for age were replaced by the mean age of all subjects. The original phenotypic value (triglyceride level) was log transformed prior to the analysis, as did by the original authors.

In the population of the Dallas Heart Study, the minor allele frequency of rare variants ranges from 0.014% to 37.9%. Most of the sequence variants have MAF less than 1%. Therefore, the data contain many rare variants as well as a few common variants.

Since there are three genes, variants within the same gene are considered in one group. The way of grouping variants can be arbitrary. Variants may be grouped based on the minor allele frequencies or predicted biological functions [26]. We may also define groups based on physical locations of the rare variants. It is reasonable to analyze the variants in the same gene as one group because variants in the same gene may work systematically as a unit.

First, we analyzed the rare variants one group (gene) at a time. The adaptive ridge regression tests the group effect of variants in the same gene. The single group model is
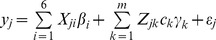
(21)where the six covariates represent the intercept (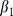), age effect (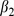), gender effect (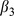), and three effects for the race (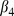,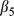 and 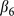). Note that there are four ethnic groups, but only three estimable effects, which explains why we have three fixed effects for the race factor alone. The number of markers 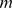 takes 88, 94 and 100, respectively, for the three genes. We reported the empirical *p*-value for each gene (group) drawn from permutation analysis (1000 permuted samples) along with the theoretical *p*-values from 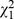 and 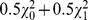 distributions. In the permutation analyses, we kept the marker genotype data intact but reshuffled the phenotype along with the six covariates (fixed effects) across all the subjects. This permutation analysis only destroyed the association between the phenotype and the markers and did not destroy the association between the phenotype and the covariates. The estimated parameters along with the test statistic and the *p*-values are listed in Table 1 for the adaptive ridge regression method. Genes *ANGPTL3* and *ANGPTL4* had *p*-values smaller than 0.05, and thus rare variants of these two genes are collectively associated with triglyceride level. Variants of gene *ANGPTL5* are not associated with the trait at all because the *p*-value is 1.00. Estimated individual marker effects will be reported later when the joint analysis of three genes are reported. We also analyzed the three genes separately (one gene at a time) using BhGLM [26]. The results of BhGLM are listed in Table 2 and they are similar to our ARR analysis. Genes *ANGPTL3* and *ANGPTL4* are strongly associated with the triglyceride level but gene *ANGPTL5* is not.

**Table 1:** Parameters of three genes of the Dallas Heart Study estimated separately using the ARR method proposed in this study.

Parameter	*ANGPTL3*	*ANGPTL4*	*ANGPTL5*
Intercept ( 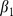 )	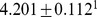	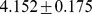	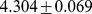
Age ( 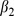 )	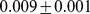	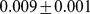	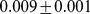
Gender ( 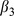 )	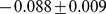	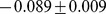	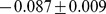
Race 1 ( 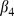 )	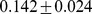	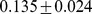	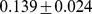
Race 2 ( 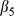 )	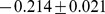	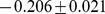	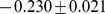
Race 3 ( 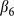 )	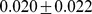	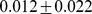	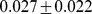
Residual variance ( 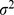 )	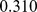	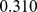	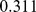
Genetic variance ( 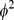 )	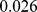	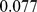	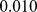
Likelihood ratio test ( 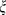 )	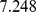	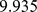	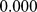
Theoretical *p*-value ( 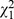 )^2^	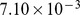	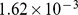	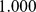
Theoretical *p*-value ( 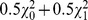 )^3^	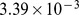	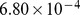	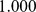
Empirical *p*-value (permutation)^4^	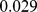	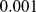	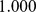

1 The numbers after 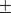 for the six fixed effects are the standard errors.

2 The theoretical *p*-value (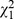) for each gene was calculated using a threshold of 3.84 for the test statistic.

3 Theoretical *p*-value (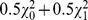) for each gene was calculated using a threshold of 2.71 for the test statistic.

4 The empirical *p*-value (permutation) was calculated using a threshold drawn from the permutation study.

**Table 2:** Parameters of three genes of the Dallas Heart Study estimated separately using the BhGLM method.

Parameter	*ANGPTL3*	*ANGPTL4*	*ANGPTL5*
Intercept ( 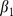 )	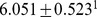	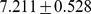	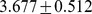
Age ( 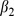 )	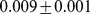	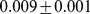	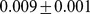
Gender ( 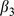 )	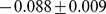	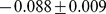	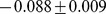
Race 1 ( 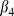 )	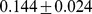	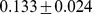	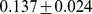
Race 2 ( 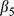 )	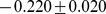	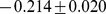	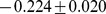
Race 3 ( 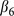 )	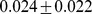	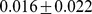	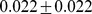
Residual variance ( 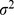 )	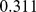	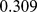	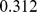
Overall score ( 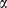 )	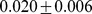	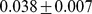	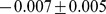
Wald test ( 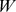 )	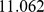	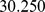	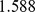
Theoretical *p*-value ( 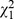 )^2^	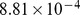	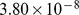	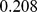
Empirical *p*-value (permutation)^3^	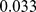	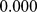	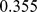

1 The numbers after 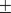 for the six fixed effects are the standard errors.

2 The theoretical *p*-value (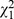) for each gene was calculated using a threshold of 3.84 for the test statistic.

3 The empirical *p*-value (permutation) was calculated using a threshold drawn from the permutation study.

**Figure 1:**
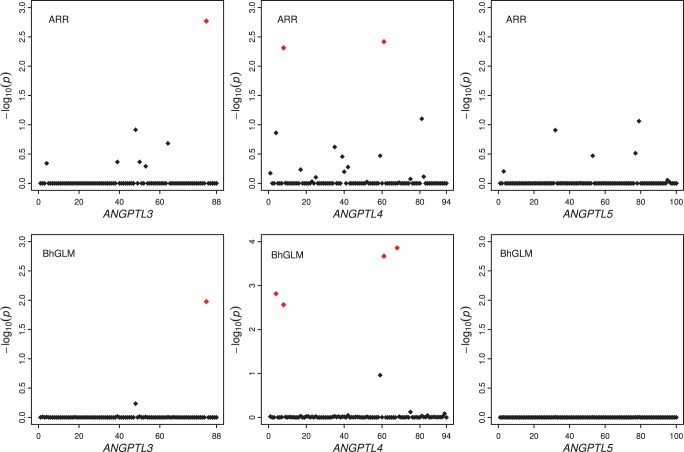
Significance level for each marker in *ANGPTL3*, *ANGPTL4* and *ANGPTL5* generated from the joint analyses. *P* value is shown on the –log_10_ scale. The top panels show the result of the adaptive ridge regression (ARR) analysis and the bottom panels show the results of the Bayesian hierarchical generalized linear model (BhGLM) analysis. The red dots represent variants with *p*-values smaller than 0.05, i.e., 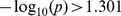.

We now report results of joint analysis for the three genes in the Dallas Heart Study. First, we used the adaptive ridge regression method to analyze the three genes jointly. This time, we have four hypotheses to test. The overall test for all three genes, 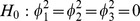, and a test for each gene, i.e., 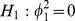, 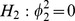 and 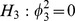. The *p*-value of each test was calculated using the permutation generated empirical threshold value of the corresponding test statistic (1000 permuted samples) and the theoretical *p*-values from 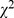 and mixture 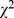 distributions. The results are listed in Table 3. First, the estimated parameters of the joint analysis regarding the model (e.g., fixed effects and the residual error variance) are similar to the separate analyses shown in Table 1. The estimated gene specific parameters and the *p*-values showed that genes *ANGTPL3* and *ANGTPL4* are associated with triglyceride level but *ANGTPL5* is not. The overall test for the three genes is also significant. This time, we also report the *p*-value for each individual rare variant, as shown in Figure 1 (the top panels). In fact, it is the 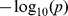 value that is plotted against the markers. One marker (8357_non_coding) in gene *ANGPTL3* is significant (*p<*0.05 and 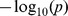
>1.301). Two rare variants (1313_E40K and 8191_R278Q) in *ANGPTL4* are significant. No variants are significant in gene *ANGPTL5*.

**Table 3:** Parameters of three genes of the Dallas Heart Study estimated jointly using the ARR method proposed in this study.

Parameter	*ANGPTL3*	*ANGPTL4*	*ANGPTL5*
Gene specific information			
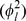 Variance component	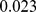	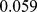	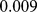
Likelihood ratio test ( 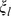 )	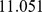	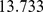	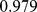
Theoretical *p*-value ( 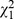 )	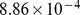	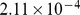	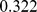
Theoretical *p*-value ( 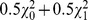 )	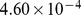	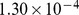	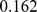
Empirical *p*-value (permutation)	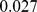	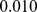	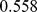
Model information			
Intercept ( 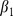 )	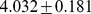		
Age ( 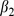 )	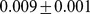		
Gender ( 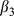 )	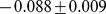		
Race 1 ( 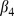 )	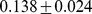		
Race 2 ( 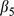 )	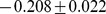		
Race 3 ( 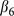 )	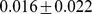		
Residual variance ( 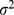 )	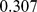		
Likelihood ratio test ( 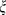 )	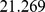		
Theoretical *p*-value ( 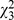 )	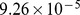		
Theoretical *p*-value ( 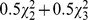 )	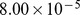		
Empirical *p*-value (permutation)	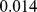		

We also analyzed the three genes jointly using BhGLM. The results of this analysis are listed in Table 4. The general conclusions are the same as the ARR analysis, *ANGPTL3* and *ANGPTL4* are associated with triglyceride level but *ANGPTL5* is not. The plot of 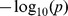 against the markers for the BhGLM analysis is presented in Figure 1 (bottom panels). The same variant (8357_non_coding) in gene *ANGPTL3* is also significant here. The two significant rare variants (1313_E40K and 8191_R278Q) detected for gene *ANGPTL4* with the ARR analysis are also significant in the BhGLM analysis. In addition, two more rare variants (1175_Intronic and 8279_P307P) are detected by the BhGLM analysis. Each of the two additional rare variants is closely linked to one of the previously detected ones by the ARR analysis. The rare variant named 1313_E40K, detected by both the ARR and BhGLM methods, was also found to be significantly associated with plasma triglyceride level in different population-based studies (Dallas Heart Study, Atherosclerosis Risk in Communities Study and Copenhagen City Heart Study) [6]. The 1313_E40K carriers had lower triglyceride level than the non-carriers.

**Table 4:** Parameters of three genes of the Dallas Heart Study estimated jointly using the BhGLM method.

Parameter	*ANGPTL3*	*ANGPTL4*	*ANGPTL5*
Gene specific information			
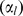 Group effect	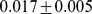	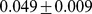	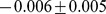
Wald test ( 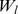 )	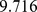	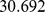	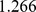
Theoretical *p*-value ( 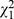 )	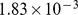	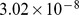	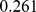
Empirical *p*-value (permutation)	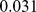	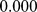	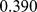
Model information			
Intercept ( 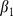 )	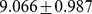		
Age ( 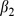 )	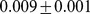		
Gender ( 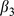 )	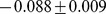		
Race 1 ( 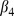 )	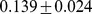		
Race 2 ( 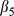 )	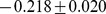		
Race 3 ( 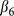 )	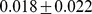		
Residual variance ( 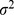 )	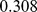		
Wald test ( 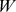 )	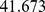		
Theoretical *p*-value ( 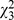 )	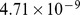		
Empirical *p*-value (permutation)	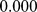		

The analyses of Dallas Heart Data showed that the method is not sensitive to the number of groups, because results of separate and joint analyses are much the same. Because ridge regression is a random model approach, the method is also not sensitive to the number of markers within each group. In fact, the number of markers of each group can be more than the sample size. This is the beauty of the random model approach, which the fixed model lacks.

### Simulation Studies

Instead of using population genetics models to generate the genotype data, we used the sequence data from the Dallas Heart Study for the simulation studies without making any assumption about the rare variants. The real sequence variants are believed to be more appropriate in the simulations [16], [26]. The covariates such as age, gender and race were included in the model. The effects of the fixed effects (including the intercept) and the residual error variance used to generate the data took the estimated values obtained from the ARR analysis for gene *ANGPTL4*. The marker genotypes took the genotypes of the 94 variants of this gene only. The purpose of the simulation study is to evaluate the empirical Type I error and statistical power. Therefore, we only used the single gene model to evaluate the Type I errors and powers of the ARR method and BhGLM for comparison.

#### Simulation of Type I Error Rate

The phenotypic value for each of the 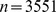 subjects was generated using the following model,
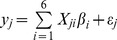
(22)where the 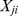
's are covariates of the Dallas Heart Study, the 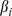
's are fixed effects estimated in the analysis of *ANGPTL4* and 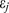 was simulated from a 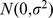 distribution with 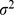 taking the value estimated from the analysis of gene *ANGPTL4*. This model assumes zero effects for all the 94 variants and thus any association of the variants is a false positive. The simulated data were then subject to the same analysis as the real data described early with all the 94 variants included in the model. For the ARR analysis, we used two criteria to evaluate the Type I error rate. In the first criterion, we choose 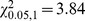 as the threshold value for the likelihood ratio statistic, above which the gene (group of variants) was declared as significance. The threshold value of the likelihood test statistic for the second criterion was 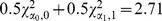, where 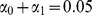. The simulation was replicated 1000 times. Under each criterion, the number of replicates whose test statistics reached the corresponding threshold was counted as the number of false positives. This number divided by 1000 is the actual (observed) Type I error rate. If the observed Type I error rate is below 0.05, we then conclude that the Type I error rate is under control (may be conservative). The criterion that is closer to 0.05 should be recommended.

**Figure 2:**
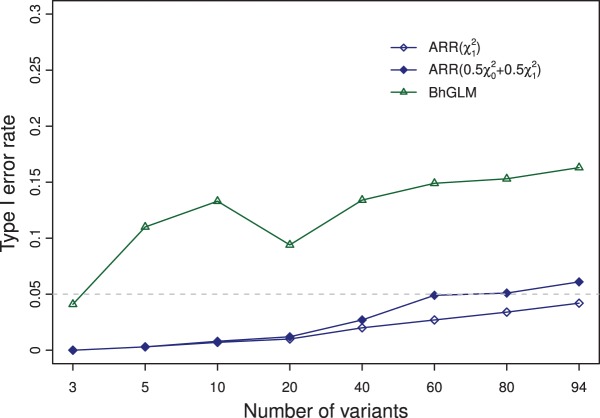
Type I error rates of the ARR and BhGLM methods obtained from the simulation studies. The Type I error rate of the Bayesian hierarchical generalized linear model (BhGLM) method was calculated using a threshold of 3.84 for the Wald test statistic. The Type I error rates of the adaptive ridge regression (ARR) method were calculated using thresholds of 3.84 and 2.71, respectively, corresponding to the 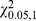 and 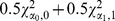 criteria (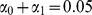).

We suspected that the actual Type I error rate is related to the number of rare variants included in the model. Therefore, we evaluated the Type I errors under different numbers of variants included, starting from 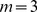 and progressively increased to 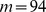. Under each model size (*m*), 1000 replicated simulations were conducted and the actual Type I error rate was observed. Figure 2 shows the observed Type I error rate plotted against the number of rare variants included in the model for the ARR method along with the BhGLM method [26]. For the BhGLM method, the test statistic was the Wald test statistic and the threshold value for the Wald test is also approximated by 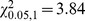. From Figure 2, we can see that the actual Type I error rate is indeed related to the model size, large models tend to give higher Type I error rates. However, according to 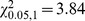 , the Type I error rate for the ARR method is under control (all below the expected 0.05 probability). Therefore, this criterion may be too conservative. For the 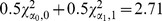 criterion, the observed Type I error rate is much closer to the expected 0.05 probability. When 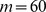 and 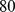, the Type I error rates are exactly 0.05. However, when *m* reaches 94, the observed Type I error rate is slightly higher than the 0.05 probability. The observed Type I error rate for the BhGLM method, however, is out of control for all model sizes examined except when only 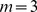 variants were included in the model.

Our conclusions from this simulation experiment are (1) the 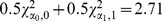 criterion is recommended for the likelihood ratio test (in the ARR analysis), (2) the 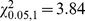 criterion for the likelihood ratio test is over conservative and may be more preferable to some investigators (in the ARR analysis) and (3) the 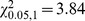 criterion for the Wald test statistics is too liberal (out of control for the Type I error rate) in the BhGLM analysis. To control the Type I error rate under 0.05 for the BhGLM, the threshold level should be further increased. The Type I error analysis is useful if permutation analysis is not performed. We realized that BhGLM allows users to choose their own prior distribution. The program also provides a set of default priors. We simply used the default priors, which may partly explain the high Type I error rates.

#### Simulation of Empirical Power

Again, we used the 94 variants of *ANGPTL4* as the true genotype data for the power analysis. The estimated fixed effects and effects of the 94 variants for *ANGPTL4* from the ARR analysis were used as the true values for the power analysis. The residual variance will determine the proportion of the phenotypic variance explained by the rare variants. Recall that the linear model for *ANGPTL4* is
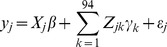
(23)where 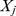 and 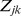 were chosen from gene *ANGPTL4* from the Dallas Heart Study, 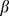 and 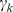 took values estimated from the ARR method, and 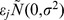 is the residual error with variance 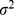. The genetic value for individual *j* is defined as



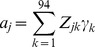
(24)The genetic variance is defined as the variance of 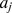 across all individuals, as shown below,
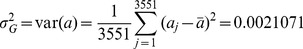
(25)


**Figure 3:**
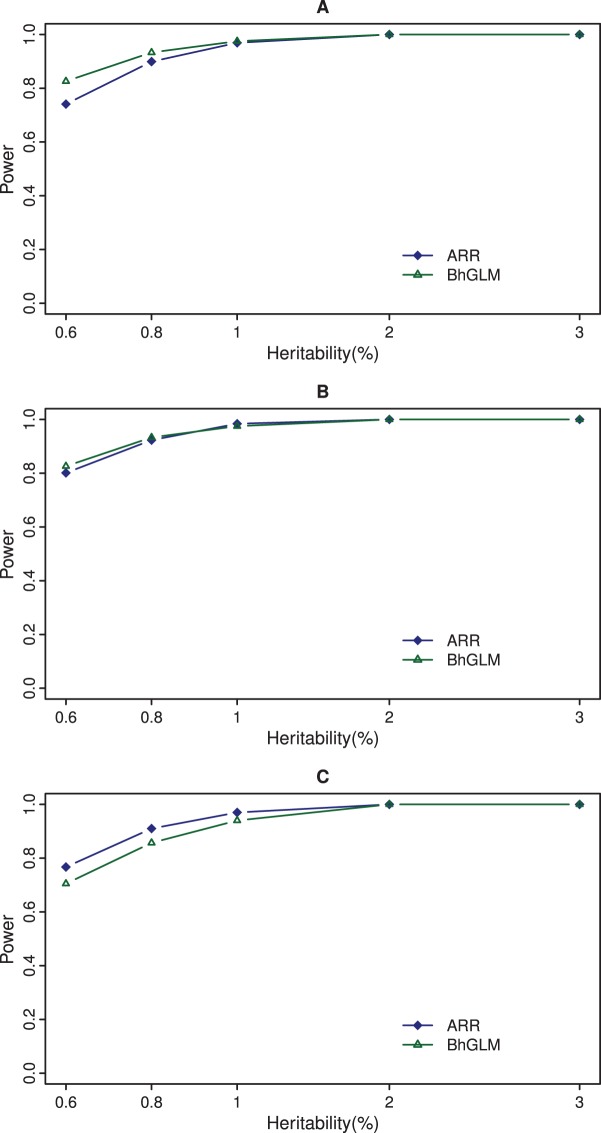
Power comparison between ARR and BhGLM at significance level of 0.05. The top panel (A) gives the powers of the adaptive ridge regression (ARR) and the Bayesian hierarchical generalized linear model (BhGLM) evaluated at the threshold of 3.84. The panel in the middle (B) shows the powers of ARR and BhGLM evaluated at the threshold 2.71 for ARR and 3.84 for BhGLM. The bottom panel (C) shows the powers of ARR and BhGLM using thresholds of 3.45 and 9.78, respectively, to control the 0.05 Type I error rate.

The total phenotypic variance is
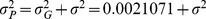
(26)in which the variance due to the covariates (fixed effects) has been removed. The heritability of the trait is



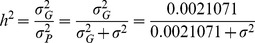
(27)We choose several different values of 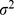 to control the heritability at the following levels, 0.6%, 0.8%, 1%, 2% and 3%. At each level of the heritability, we calculated 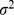 and used 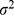 to simulate a random residual error to add to the fixed effect and the genetic effect to generate a phenotypic value 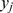. At each level of the heritability, the simulation was replicated 1000 times. The empirical statistical power was then obtained by counting the proportion of the replicated samples that are significant over the 1000 replicates. For the ARR method, three criteria were used to determine the threshold values for the likelihood ratio test statistic. They are 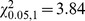, 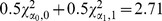 and 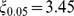. The last one, 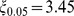, was obtained from the simulation experiment in the section of Type I error rate study. To compare the power of the ARR analysis with that of the BhGLM analysis, the same datasets were also analyzed using the BhGLM program. The power of BhGLM was determined using two criteria, 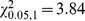 for the Wald test and 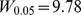 obtained from the null model simulation study (Type I error rate study). We knew that using the 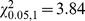 criterion would overestimate the power for the BhGLM method because the actual Type I error rate for the BhGLM analysis was much higher than 0.05. The power analysis showed that using the theoretical threshold 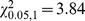, the BhGLM method appears to be more powerful than the ARR method (see Figure 3, panel a). However, the ARR analysis based on 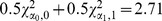 has almost the same power as BhGLM at different levels of heritability (see Figure 3, panel b). When the empirical thresholds are used (drawn from the Type I error rate study), the ARR method is more powerful than the BhGLM method (see Figure 3, panel c). A permutation generated threshold for the BhGLM method should be used in real data analysis because the Type I error rate cannot be controlled using the theoretical threshold.

## Discussion

**Figure 4:**
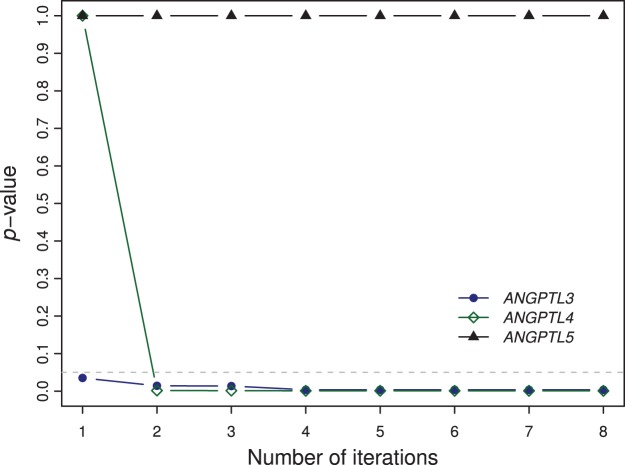
Changes of *p*-values of the three genes during the iteration process in the separate analysis. The *p*-values of the three genes in separate analysis using adaptive ridge regression (ARR) are plotted against the iteration process. P-values are calculated based on 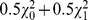 distribution.

The adaptive ridge regression method was developed based on the original ridge regression [27]. The purpose of the adaptation is to selectively weigh each rare variant based on its size, denoted by 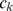 for the *k*th rare variant, so that the overall genetic variance 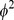 is not “diluted” by the non-associated variants. The adaptive ridge regression requires just a few iterations to converge. Figure 4 shows the iteration process of the *p*-values calculated from the 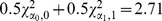 criterion for the three genes (*ANGPTL3*, *ANGPTL4* and *ANGPTL5*) analyzed separately by the ARR method. For gene ANGPTL3, the *p*-value of the initial step (ridge regression without adaptation) is greater than 0.05. Just one step of adaption, the *p*-value has dropped to the 0.05 significance level. For gene *ANGPTL4*, the *p*-value of the initial step (ridge regression without adaptation) is already lower than the 0.05 probability. Further iterations continue to drop the *p*-value. For gene *ANGPTL5*, the *p*-value is very high and remains high after iterations. This figure clearly shows the necessity of the adaptive steps for rare variant detection.

One reviewer brought a recent publication to our attention [41]. The method is called sequence kernel association test (SKAT). After reading this paper, we agreed that our approach is similar to SKAT. However, SKAT only gives the score test and no parameter estimation is provided. This explains why SKAT is fast computationally. There are three major advantages of the adaptive ridge regression. First, a high score test does not mean the effects are large. It may be caused by small effects but large sample size. The score test cannot tell the difference. Our method not only provides a test but also an estimate of the group variance. We can provide a total proportion of the phenotypic variance contributed by the rare variants. Secondly, we introduced an adaptive step to the original ridge regression. This step plays the role of “weighting” of the SKAT method but it can “homogenize” the effect of each rare variant within a group. The ridge regression performs better under the “homogenized” rare variant effect assumption. Thirdly, our method works for both rare and common variants. However, the SKAT method was particularly designed for rare variants because the “weights” for the common variants will be almost zero (excluded from the model), according to the authors of that paper. There is a possibility to use the score test under our adaptive ridge regression framework. The estimation procedure will remain the same, but we may simply replace the likelihood ratio test by the score test. The “weights” obtained from the adaptive ridge regression will be used in the score test. This needs to be further investigated.

We did not compare the ARR method with other rare variant detection methods other than the BhGLM method. The reason for this is that Yi and Zhi [20] already compared BhGLM with many other methods and showed that BhGLM outcompeted all of them. Given the fact that our method is more powerful than BhGLM (simulation study), we concluded that the ARR method is also more powerful than the other methods. The BhGLM program provides a set of default priors, which were used in this study. Users do have the option to choose their own priors. If different priors were chosen, the power of the BhGLM may change slightly (in either direction). The default priors provided by Yi and Zhi (2011) were drawn from extensive simulation studies and should be quite robust. It is difficulty to choose the optimal set of priors in simulation studies. However, it is easy to choose the optimal prior set in real data analysis. We need a criterion to evaluate the priors. Statistical power is not a viable criterion in real data analysis because the true rare variant effects are not known. The mean squared error (MSE) via cross validation may be a viable choice for the criterion. This requires further investigation. Our ARR method is a maximum likelihood approach, equivalent to uniform priors for all variances. In theory, we can also assign the variances to other priors to improve the power. This deserves further investigation.

The adaptive ridge regression method has been shown to be the Lasso [33] estimation if the Lassos parameter 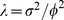 is predetermined by the investigator. Our new contribution is to estimate 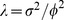 using estimated variance components. This approach has provided a new way to select the shrinkage factor 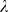 based on data. In the original Lasso method, the author used cross validation to determine the shrinkage factor. With the new method, the Lasso parameter is estimated from the data and thus has eliminated the cross validation step. The extension of the ARR to multiple groups of rare variant detection is conceptually similar to the group Lasso method [42], [43] in which different groups have different Lasso parameters, as given by 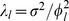. This idea has a general application to detection of multiple groups of variants as well as their interactions (epistatic effects). The current methods of rare variant detection have not been able to detect interactions between two groups of rare variants. The Dallas Heart Study dataset contains three genes (groups). Our next project will be analyzing the three pairs of group interactions among the three genes. Gene *ANGPTL5* has no effect on triglyceride level. However, it may interact with other two genes. The full model will include three group variances plus three variance components of the interaction.

The Lasso method itself may not be perfect for all data. It may work for some data but not work for other data. Using the adaptive ridge regression approach, we may modify the shrinkage factor through different choice of the constraint. For example, the constraint of 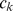 given by Grandvalet [28] is 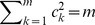. This constraint determines the level of shrinkage. An obvious extension may be 
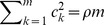
, where 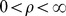 is another factor we can use to control the strength of the shrinkage. Our adaptive ridge regression is equivalent to 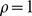, a special case of the general method.

The new method is developed for continuous traits under the linear mixed model framework [30]. In many situations, the trait of interest may be a binary trait. The generalized linear mixed model (GLMM), which is an extension of the linear mixed model, can be used to analyze the association of multiple rare variants and a binary trait. This extension is very straight forward because the methodology of GLMM has been well established. The simple extension includes the adaptive steps.

Finally, we performed all the analyses using an R program. The R package is called Adaptive Ridge Regression (ARR) which can be downloaded from the authors' personal website: www.statgen.ucr.edu.
